# What do we know about what happens to myometrial function as women age?

**DOI:** 10.1007/s10974-012-9300-2

**Published:** 2012-05-30

**Authors:** Sarah Arrowsmith, Hayley Robinson, Karen Noble, Susan Wray

**Affiliations:** Department of Cellular and Molecular Physiology, Institute of Translational Medicine, University of Liverpool, Liverpool, UK

**Keywords:** Myometrium, Contraction, Pregnancy, Non-pregnant, Maternal age

## Abstract

Much has been written about the effects of aging on reproductive function, especially female fertility. Much less is known about how aging may affect the contractility of the smooth muscle within the uterus, the myometrium. The myometrium is active through a woman’s entire life, not just during pregnancy. Here we will discuss briefly the contractile functions of the uterus and the changes it undergoes throughout the stages of a woman’s life from menstruation and the menopause, before evaluating the evidence for any changes in myometrial contractility and responses as women age, with a particular focus on women of advanced maternal age. We present original contractility analysis for the widest data set for human myometrium so far examined, and determine inherent spontaneous activity as well as responses to depolarisation and stimulation with oxytocin. Our data show that in the non-pregnant state there is a significant decrease in contractility for both spontaneous and depolarised-induced contractions, with age. We suggest that muscle atrophy and down regulation of Ca channels may account for this. Interestingly in pregnant myometrium we found a wide range of contractile ability between women and little evidence for decreased spontaneous activity between the ages of 25–40. Oxytocin responses appear to be more affected by aging, a finding that is consistent with previously reported clinical findings, and may partly be the result of membrane lipids such as cholesterol, increasing as women age. The marked differences between the age-related decline of force beyond age 30 in non-pregnant uterus, and the lack of difference in the pregnant state over this period, shows that the uterus retains its ability to respond to gestational hormones. The growth of the pregnant uterus and increase in content of myofibrillar proteins, may abolish any previous age-related force deficit. This finding is consistent with what is apparent for postmenopausal women in their 50s and 60s; that with the appropriate hormonal stimulation the uterus can allow an embryo to implant, and then without further intervention, carry the foetus to term. It is tempting therefore to speculate that unlike other well documented declines in female reproductive functions with age, the myometrium remains able to function into a woman’s 7th decade.

## Introduction

The purpose of this article is to consider how aging may affect uterine function, with a focus on contractility. The smooth muscle of the uterus, the myometrium, is active throughout a woman’s life, not just during labour and delivery. The mechanism generating the contractile activity and its physiological regulation are reasonably well understood and have been described elsewhere (Aguilar and Mitchell 2010; Noble et al. 2006; Shmigol et al. 1999; Wray 2007; Horowitz et al. 1996; Szal et al. 1994). Here we will discuss briefly the contractile functions of the uterus and the changes it undergoes at menstruation and the menopause, before evaluating the evidence indicating changes in myometrial function as women age, with a particular focus on women of advanced maternal age.

### The myometrium, menstruation and menopause

The first contractions felt by a woman are those occurring once menstruation has started, possibly those giving rise to the uterine cramping (dysmenorrhea) often associated with menstruation. Non-pregnant myometrium has been shown to undergo different patterns of contractility during the menstrual cycle; one being ‘focal and sporadic bulging of the myometrium’, (Togashi 2007; Togashi et al. 1993) giving rise to sustained contractions and the other, rhythmic, ‘wave-like’ contractions, sometimes called uterine peristalsis, which are thought to aid in the sloughing of the endometrium (Bulletti et al. 2000; de Vries et al. 1990; Lyons et al. 1991). The female steroid hormones change during the menstrual cycle and influence the pattern of myometrial activity in women and other animals (Wray and Noble 2008). These hormones will also control levels of ATP and other metabolites needed for contraction (Crichton et al. 1993; Wray and Tofts 1986) and can affect excitability (Parkington et al. 1999). Recordings of uterine pressures in the non-pregnant uterus as well as MR imaging have shown that the pattern of myometrial activity, such as the direction of contraction propagation throughout the uterus varies with the different phases of the menstrual cycle (Bulletti et al. 2000; Kunz and Leyendecker 2002; Nakai et al. 2003; Togashi 2007), although diurnal variations, have not been observed (Kido et al. 2006). The pattern of contractile activity in the non-pregnant uterus is closely related to uterine function. Thus antegrade contractions, that is contractions propagating from the fundus towards the cervical end of the uterus, favours forward emptying or discharge of uterine content i.e. menstrual blood, (Lyons et al. 1991) whilst cervico-fundal contractions aid in sperm transport or possibly in retention of iron for example, following blood losses at menstruation (Kunz and Leyendecker 2002). In the pregnant uterus, these retrograde contractions may also have roles in the maintenance of early pregnancies within the uterine cavity (de Vries et al. 1990), but possibly also in causing endometriosis, as menstrual debris enters the peritoneal cavity.

The menopause marks another hallmark in a woman’s life. With the gradual decline in hormonal secretion from the ovaries, up to the point when the ovaries cease to function, the menopause is characterised by a loss of regular menstruation and eventually permanent cessation of menses (Burger et al. 2002). One might assume that following the menopause, uterine activity ceases. However, rhythmical myometrial contractions have been seen by ultrasound examination in postmenopausal women (de Vries et al. 1990) and one previous study has reported low levels of spontaneous activity in strips from human post-menopausal myometrium in vitro (Domali et al. 2001).

### Myometrial function in pregnancy and labour

The myometrium in pregnancy must change from a period of relative quiescence during pregnancy to an actively contracting organ, for labour to progress and mediate delivery of the foetus and placenta. Morphologically the myometrium undergoes many changes and adaptations in order to successfully ‘house’ the products of conception and accommodate a growing foetus. This involves hypertrophy and hyperplasia of the myocytes and mechanisms to inhibit the build up and spread of contractions, which include high levels of progesterone and low expression of gap junctions to prevent the synchronisation and spread of Ca signals (Garfield 1988; Thilander et al. 1993; Bernal 2001). The shift to contraction rather than relaxation of contractility heralds the onset of labour, although the mechanism of this switch is unknown (Price and Bernal 2001). Little is known therefore about why some women go beyond term (Arrowsmith et al. 2011) although it can be noted that poor contractility has recently been identified as one of the underlying causes, and the risk of post term pregnancies increases with maternal age (Arrowsmith et al. 2012).

From the onset of menstruation to menopause, a woman’s natural reproductive life is approximately 40 years. With the advance of assisted reproductive technologies however, the boundaries of the reproductive window are continuously being pushed higher. As a result, the number of women of peri- menopausal age and beyond, who are pregnant is steadily increasing. Thus the question of how well the myometrium can switch from the non-pregnant to the highly muscular pregnant state, and how it performs in aiding conception, maintaining the foetus and then delivering it, is now beyond academic interest alone.

Given the plethora of studies examining the effect of older maternal age on the success of IVF and oocyte donor transfer, the function of the myometrium and how it may be altered in the older gravida has received minimal attention. There is a paucity of data examining the physiological changes within the human uterus with aging and only a handful of studies using animal models exist (Maurer and Foote 1972; Mulholland and Jones 1993; Rigby et al. 2001). How age impedes the ability of the myometrium to support and adapt to the growing foetus and participate in active labour is still under debate. Much of what is known about myometrial function and the implications of aging on the human uterus has been gleaned from observational data on rates of caesarean section delivery and labour outcomes.

### Myometrium and aging: indications from pregnancy and labour outcomes in the older mother

Many studies have shown that women of older maternal age have a higher rate of caesarean section delivery compared their younger counterparts (Bianco et al. 1996; Cnattingius et al. 1998; Gordon et al. 1991; Kirz et al. 1985; Main et al. 2000; Schoen and Rosen 2009; Smith et al. 2008) however, it is not yet known whether this elevated rate is associated with an underlying biological cause of aging on the uterus and thus due to changes in its function or if it is due to a physician or maternal choice (Bell et al. 2001). For example, older women more commonly have a number of pregnancy complications which itself influences the rate of CS delivery. These complications include medical illnesses such as pre-eclampsia, hypertension and gestational diabetes (Bianco et al. 1996; Le Ray et al. 2012; Yogev et al. 2010; Ziadeh and Yahaya 2001). However, it has been reported that women of older age without such conditions, still have more CS deliveries than their younger counterparts (Adashek et al. 1993; Kirz et al. 1985). It has been suggested by some that, given that many women of advanced age will have become pregnant as a result of assisted reproduction, older women may have made more significant emotional and financial investments into their pregnancies and these pregnancies are often considered to be ‘extra precious’ thereby creating more anxiety and concern on behalf of the physician and the mother. With this in mind, many women (and their practitioners) may therefore elect for a caesarean delivery to avoid potential difficulties with a normal vaginal delivery. In this instance, preference of delivery method would have a large bearing on caesarean delivery rates in the older population and hence would not reflect a true ‘biological need’ in the older parturient.

Studies examining labour characteristics have shown that length of labour and incidence of prolonged labour does vary by maternal age. In a study by Greenberg et al. (2007), older women were shown to persistently have longer first and second stages of labour and higher rates of prolonged first and second stage. In a UK study, Smith et al. (2008), examined the association of maternal age on rates of intrapartum caesarean section and outcome of labour using data collected in Scotland over a 25 year period. Older age in first pregnancy was associated with both an increased risk of caesarean section and increased duration of labour. Moreover, the need for operative vaginal deliveries was also higher in women of advanced age and these findings were shown not to be explained by co-morbidities such as obesity or gestational diabetes. One hypothesis proposed for these findings is that with increasing age, the myometrium is less effective or less responsive to uterotonic agents such as oxytocin or prostaglandins thereby indicating a direct effect of aging on myometrial function (Greenberg et al. 2007). This hypothesis is supported by Main et al. (2000) who indicated an increasing need for oxytocin-augmentation in women of older age and suggested that this effect of aging was gradual and a continuous process (Main et al. 2000).

### The effect of aging on myometrial function

Whilst inferences about myometrial function in the elderly gravida can be obtained from examining pregnancy and labour outcomes, they are limited in that they do not measure the intrinsic contractile abilities of the myometrium per se. In their 2008 study, Smith et al. 2008 performed an in vitro study examining the contractility of strips of myometrium from women of differing age up to age 46 and concluded myometrial function decreased with increasing maternal age. However to date no study has looked at the function of myometrium from women much over 40, and certainly not for women in their 50s or even older, nor at the activity of the non-pregnant uterus with age. We have therefore examined contractility in over 100 women (aged 25–72), and given the clinical data and hypothesis that older myometrium is less responsive to agonists (Main et al. 2000), we also examined myometrial contractility stimulated with oxytocin.

## Methods

### Direct measurements of human myometrial contractility with age

Women were recruited from the Liverpool Women’s Hospital NHS Foundation Trust, UK after giving informed written consent to a biopsy, in an ethically approved study. Myometrium was obtained from women aged 25 or older, either undergoing elective caesarean section (CS) with a single baby, at term (38–40 weeks gestation), not in labour (mean maternal age, 33; range 25–42 years, *n* = 80), or following hysterectomy (mean maternal age, 44; range 27–63 years, *n* = 24). Indications for CS included; previous CS, breech presentation or previous difficult vaginal delivery. Indication of hysterectomy included menorrhagia, irregular bleeding, pelvic pain or prolapse. Clinical details of the women in this study are presented in Table 1. Women were not recruited if they had any serious medical complications or used medication likely to affect myometrial activity (e.g. antihypertensive medication). Biopsies were obtained from the upper edge of the lower uterine segment incision during CS, immediately after delivery of the baby (Luckas and Wray 2000). Non-pregnant biopsies were obtained from the corresponding area of the normal uterus at hysterectomy. All biopsies were immediately placed in Hanks balanced salt solution (Sigma Aldrich, Poole, UK) at 4 °C and transferred to the Smooth Muscle Research Laboratory, University of Liverpool.

**Table 1:** Demographics and clinical details of women used in this study according to pregnancy status

Pregnant women (*n* = 80)	
Age (years), mean (SD)	33 (5.4)
Gestational age (days), mean (SD)	274 (2.8)
BMI (kg/m^2^), mean (SD)	28.7 (6.3)
Birthweight (grams), mean (SD)	3,532 (516)
Primiparous, *n* (%)	11 (13.9)
Reason for CS, *n* (%)	
Previous CS	54 (67.5)
Breech	20 (25.0)
Previous difficult pregnancy	5 (6.3)
Placenta previa	1 (1.3)
Non-pregnant women (*n* = 24)	
Age (years), mean (SD)	44.3 (10.4)
BMI (kg/m^2^), mean (SD)	25.6 (2.9)
Reason for CS, *n* (%)	
Menorrhagia	12 (50.0)
Prolapse	3 (12.5)
Irregular bleeding	1 (4.2)
Pelvic pain	1 (4.2)
Not stated	7 (29.2)

Isometric tension recordings were performed on strips of myometrium within 12 h of biopsy excision; 5 mm long, 2 mm wide, and 1 mm thick, dissected under stereomicroscope so that the longitudinal axis was aligned with the direction of the muscle fibres. Full details of the methodologies can be found in previous publications (Heaton et al. 1993; Kupittayanant et al. 2002; Taggart et al. 1997). Briefly, the strips were mounted, secured by aluminium clips and under a resting tension of 2 mN, in a 1 ml bath and superfused with physiological saline (PSS) at 1.5 ml/min, pH 7.4 and 36 °C and tension recorded (FT03, Grass transducer and Axon acquisition, Shmigol et al. 1999). Strips were left to equilibrate for 2 h to generate a steady baseline tension and regular spontaneous contractions. In most experiments response to high K^+^ (40 mM) depolarisation was tested and in some, oxytocin (10 nM) was investigated. Strips were measured and weighed to determine force per cross sectional area.

Contractility measurements were made and analysed blinded to the woman’s age. Age in whole years was that at CS delivery or hysterectomy. Force is expressed as mean ± SEM and peak amplitude (mN or relative to high K, 100 %) and the area under the curve (AUC) expressed in arbitrary units, au) calculated. Responses to oxytocin were expressed as % of previous control activity (100 %). Data are expressed as the mean ± SEM where ‘*n*’ is the number of women. Statistical and graphical presentations were generated using Origin Pro 8.5. Significance was tested with Spearman’s rank correlation coefficient test (correlation coefficient is provided as *r*).

## Results

### In vitro contractility with age

**Fig. 1:**
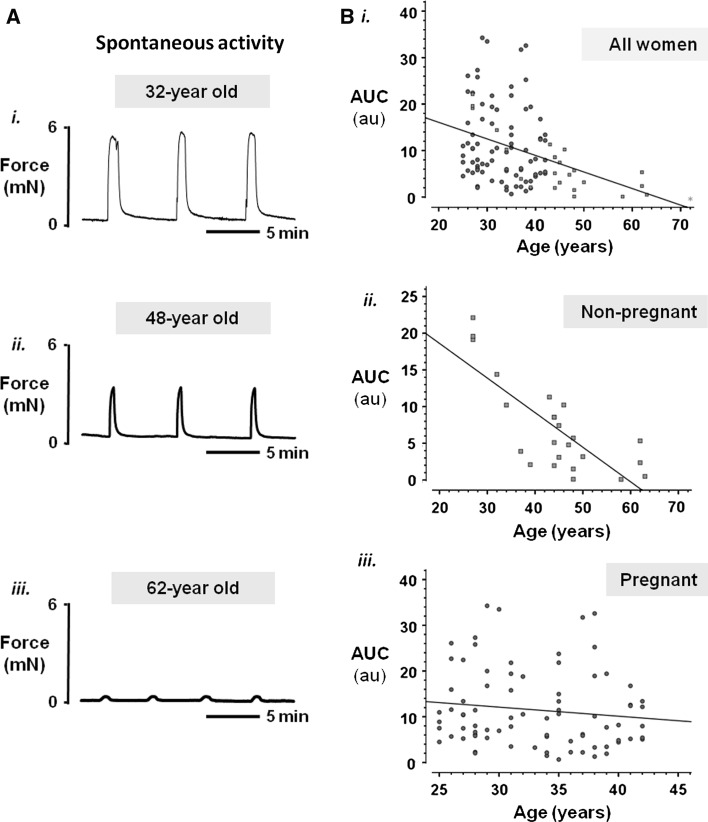
Myometrial contractility in relation to maternal age. **a** Representative isometric recordings of spontaneously contracting myometrial strips obtained from a (*i*) 32-year old, (*ii*) 48-year old and (*iii*) 62-year old woman undergoing hysterectomy. Strips were placed under a resting tension of 2 mN and superfused continually with physiological saline solution (pH 7.4) at 37 °C. **b** Plots of spontaneous contractile activity (quantified as area under the contraction curve, (AUC, in arbitary units, au) of strips of pregnant and/or non-pregnant myometrium obtained from women undergoing planned CS delivery or hysterectomy respectively, against age. Pregnant women are denoted by *blue circles* and non-pregnant women are denoted by *red squares*. *Green asterisk* was from a 72-year old woman but produced no spontaneous contractions (and was not included in the analysis). Spearman’s rank test found a significant negative correlation between integral of force and age (*i*; combined data, *r* = −0.379, *P* < 0.01, *n* = 104). (*ii*, *iii*) Plots of non-pregnant and pregnant women separately. A significant negative correlation between integral of force and age was found for non-pregnant women alone (*ii*; *r* = −0.529, *P* = 0.008, *n* = 24). In contrast, no significant correlation was found for pregnant women (*iii*; *n* = 80). (Color figure online)

Figure 1a show three original contractility recordings from the myometrium of a woman aged 32 (i), 48 (ii) and 62 (iii). All three traces are from non-pregnant women, and a very clear loss of force is apparent with increasing age. When data from all women (pregnant and non-pregnant) are plotted (Fig. 1b) this clear drop in force with age is seen (*r* = 0.379, *P* < 0.01, *n* = 104). However it can also be seen that this trend is much more a feature of the non-pregnant uterus (red squares) than the pregnant uterus (blue circles). The insets in Fig. 1b show the data separated for the two groups and while the contractility of the non-pregnant uterus remains significantly negatively correlated with age (Fig. 1bi, *r* = −0.529, *P* = 0.008, *n* = 24), significance is lost with pregnant myometrium (Fig. 1bii, *n* = 80). Indeed it can be seen that many pregnant samples from women around age 40 contracted as well as those from women around age 25. It is clear that there is much scatter on these data from pregnant women. This is unlikely to be due to gestational differences as all biopsies were obtained around term (38–40 weeks gestation). Furthermore the BMI of the women in this study and their baby birthweights were similar between age groups. Sub-analysis to control for parity by removing primiparous mothers from the dataset did not improve the scatter (data not shown). And whilst reason for C-section may influence contractility, examination of data from only women having surgery for previous CS, did not change the data spread.

**Fig. 2:**
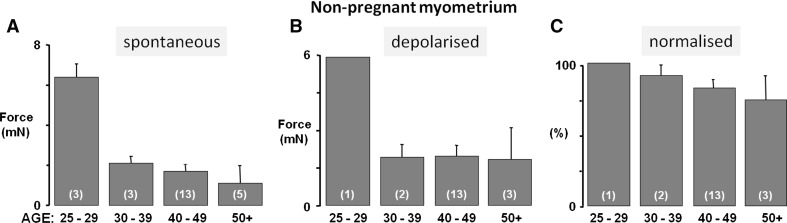
Uterine activity with age of non-pregnant myometrium. **a** Spontaneous force of contraction, **b** force of contraction under high (40 mM) K^+^ depolarisation and **c** force of contraction normalised to high K^+^ (where high K^+^ equalled 100 %), according to age group. Mean force of contraction declined with increasing age group (**a**) with little activity observed from myometrial strips from women over 50 years of age, in comparison to their younger counterparts. Similarly, maximal force achieved under high K^+^ (**b**) was reduced in samples from older women, and normalised force shown in (**c**) also declined with advancing age. Values represent means ± SEM denoted by *error bars*, values in *parenthesis* indicate *n*-numbers

**Fig. 3:**
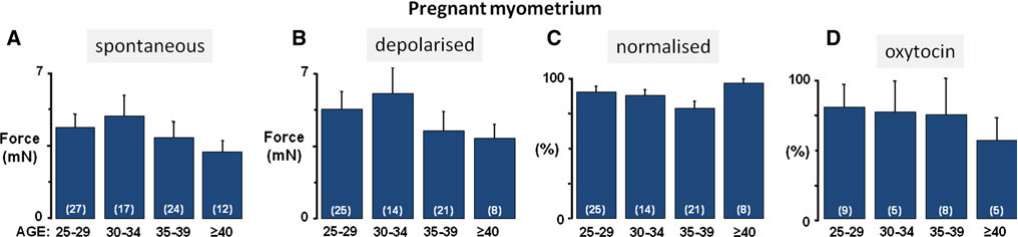
Uterine activity with age of pregnant myometrium. **a** Spontaneous force of contraction, **b** force of contraction under high K^+^ depolarisation, **c** force of spontaneous contraction normalised to high K^+^ (where high K^+^ equalled 100 %) and **d** oxytocin (10 nM)-induced percentage increase in integral of force (AUC), according to maternal age group. There was a small downward trend in mean force of spontaneous contraction with increasing maternal age group (**a**) but this was not as marked as found in non-pregnant myometrium. Maximal force achieved under high K^+^ (**b**) showed a small decrement with increasing maternal age however, normalised force shown in (**c**) did not change with advancing age, suggesting aging has little effect on myometrial function in pregnant women. Response to 10 nM oxytocin (% increase in integral of force compared to control, (**d**) declined with increasing maternal age group suggesting that changes in receptor expression or coupling to calcium entry and contraction may be reduced, especially beyond 40 years. Values represent means ± SEM denoted by *error bars*, values in *parenthesis* indicate *n*-numbers

To investigate the effects of age on the non-pregnant and pregnant uterus in more detail, we examined the effects of stimulating the uterus with high (40 mM) K^+^ which maximally activates functional voltage-gated L-type Ca channels inducing Ca entry and a large tonic contraction (Wray et al. 2001). For each tissue, spontaneous contractions were normalised to this maximal high K^+^-induced contraction to determine the degree of activation of the contractile mechanisms. Because of the smaller data sets the results have been analysed for age cohorts, as shown in Figs. 2 and 3.

### Non-pregnant myometrium

The reduction in spontaneous force with age produced by the non-pregnant uterus was striking (Figs. 1b*i*, 2a) and is also seen when L-type Ca channels are maximally activated with high K^+^ (Fig. 2b). These data suggest that there might be loss of functional muscular tissue (e.g. less muscle cells) or decreased expression of L-type Ca channels, with age (Floyd and Wray 2007; Taggart et al. 1997), although larger sample numbers are required before these suggestions can be tested.

In Fig. 2c the spontaneous contractions are normalised to the high K^+^ contraction to control for any effects of age on decreased muscle or L-type Ca expression. Relative to high K^+^, the amplitude of spontaneous activity decreases with age, suggesting that the mechanisms generating spontaneous contractions are also failing. Thus in non-pregnant myometrium we suggest that force may decline as a consequence of muscle loss and/or decreased Ca entry and thus decreased activation of the contractile proteins. Reduced levels of oestrogen with aging would be expected to cause atrophy of the myometrium, and thus explain some of the decreased strength of contractions.

There is evidence that the L-type calcium channel subunits and function are hormonally regulated, with oestrogen associated with increasing, (Batra 1987) and progesterone decreasing (Tezuka et al. 1995) calcium channel expression. Thus as oestrogen decreases with aging, levels of L-type Ca channels will be expected to fall.

### Pregnant myometrium

In pregnant myometrium as shown in Fig. 3a and b there was a small downward trend in the data for strength of spontaneous contraction and contraction induced by high K^+^ depolarisation, but this did not reach significance. As shown in Fig. 3c, the ratio of spontaneous contractions to high K^+^ depolarisation was unchanged suggesting that the activation of contraction was similar within this age range.

It had been suggested that oxytocin responses could be reduced in older women. In the present study, oxytocin (10 nM) augmented spontaneous myometrial contractions (integral of force; AUC) in all samples. Percentage increase in AUC was reduced in older women (Fig. 3d; *P* = 0.07, *n* = 28), although this did not reach significance, perhaps due to the lower number of women in this study. This suggests that either receptor expression or coupling to Ca entry and contraction may well be reduced as maternal age advances. These changes are unlikely to be accounted for by differences in the hormonal environment, as all the women were term pregnant. Lipid environment can affect oxytocin receptor function, and this is described in more detail later.

## Discussion

Our experimental data shows aging decreases uterine contractility but only to a significant extent in the non-pregnant state. Our data suggests that in the non-pregnant uterus, most of this decline may occur by the age of 35 years. Of note also, contractility although low, is maintained well into post-menopause. This is consistent with an earlier in vitro study which had found spontaneous activity in post-menopausal women (Domali et al. 2001). These latter authors also demonstrated responsiveness to endothelin 1, which could be modified by ovarian steroids. This again emphasizes that post-menopausal human myometrium remains active and responsive to hormonal environment. The reduction of ovarian steroids presumably underlies the decrease in uterine mass and reduced contractility compared to pregnant myometrium, but the uterus retains activity and the potential to respond to alterations in endocrine stimulation.

The more or less maintained force in pregnant women up to around age 40, suggests that if a woman becomes pregnant at this age, the uterus will respond to the hormonal environment of pregnancy, and increase its muscle mass, and hence contractile potential, removing any age-related differences (Wu et al. 2008). Oxytocin may have reduced efficacy in the myometrium of older women, and further studies should investigate the details of the underlying mechanisms.

### Suggested mechanisms underlying decreased contractility with age

#### Morphology

Data on how the myometrium changes with age are scarce. Whilst the morphology of endometrial tissue has been shown to change as a result of reductions in steroid hormones, how aging affects the morphology of the myometrium has not yet (to the best of our knowledge) been discussed, although elegant studies on the microanatomy the aging reproductive tract have been published (Heyn et al. 2005). Changes in the biophysical properties of the myometrium with age also appear to have been neglected. It would be interesting to examine how myocytes size, muscle mass and connective tissue content are affected by aging. We have recently found in women with age-related diabetes (type 2) that there is a small but significant decrease in uterine muscle content compared with matched non-diabetics (Al-Qahtani et al. 2012).

#### Cholesterol

Aging produces changes in metabolism including an increase in cholesterol and LDLs. Body mass index (BMI) also increases as women age. Both cholesterol and BMI have been shown to depress myometrial contractility (Zhang et al. 2007a, b), and could therefore contribute to a reduction in function.

We have previously shown that pharmacological addition of cholesterol to contracting strips of human and animal myometrium in vitro inhibits activity. Conversely, removal of cholesterol using cholesterol sequestering agents enhances contractile activity, thus in the uterus, cholesterol inhibits function. We have shown this to be due in part to effects on K channel activity (Shmygol et al. 2007; Noble et al. 2006). Furthermore, myometrial strips with high cholesterol content (measured using thin layer chromatography) contracted more poorly in the laboratory than those with a lower cholesterol content (Noble et al. 2009). We therefore investigated whether changes in myometrial membrane cholesterol may differ with increasing maternal age. We examined the levels of myometrial cholesterol content from a subset of the pregnant women within this study (*n* = 72). We found a positive correlation between maternal age and myometrial cell cholesterol (data not shown). As mentioned earlier, oxytocin receptors on the myometrial membrane are influenced by lipid content. Specifically the high affinity form of the receptor has been localised to membrane caveolae (lipid rich microdomains) (Zhang et al. 2007b; Smith et al. 2005). Thus if this environment is disturbed with aging then responses to oxytocin may also change (Gimpl et al. 2000; Gimpl and Fahrenholz 2000). Such subtle changes in environment may contribute to the decreasing response to oxytocin with age, that we found, and has been suspected clinically.

## Conclusions

In the western world, the prevalence of advanced maternal age in pregnancy for first-time mothers is increasing. Advanced maternal age is seen to be correlated with poorer obstetric outcomes which may be largely attributed to the higher incidence of high-risk medical conditions that are associated with older women. These medical risks may be associated with an aging reproductive system or alternatively an aging body or contributions from both. Does the myometrium undergo morphological changes with age and experience the aging effect as seen by declining muscular strength in other muscle types? Perhaps for older mothers there is a need for greater patience in labour to help achieve a lower rate of caesarean section? It may also be that higher doses of oxytocin are needed to help stimulate the uterus in older mothers who are progressing too slowly in labour.

We must remember that many women of older age do give birth without adverse maternal or neonatal outcomes. Moreover, pregnancy success for women over the age of 50 years has been documented following oocyte donation and sex steroid replacement and many carry their pregnancies to term. Thus in the older gravida, the function of the myometrium to expand, adapt and withstand a developing foetus would appear to be maintained. Our data support a large incremental decrease in contractility when the non-pregnant uterus is examined but only a marginal effect of age in the pregnant myometrium. Furthermore during pregnancy, there is a wide range of force produced by women at any age group, and many aged 40 or more could produce as much force as those in their 20s. Our data suggests that this may be due to the myocytes responding to the hormonal environment of pregnancy by hypertrophy and hyperplasia, and restoring any age-related deficits from the non-pregnant state. This suggestion is consistent with the uterine responses of women in their 50s and 60s to hormonal treatment for IVF and the uterus safely bearing these pregnancies to term. Thus for older women, perhaps unlike other aspects of fertility, a decline in myometrial ability is something they or their doctors can worry less about. Reducing these anxieties could of course also increase the chances of a successful vaginal delivery. We suggest further research into myometrial function with aging would be advantageous, particularly with respect to oxytocin and other uterotonics.

## References

[CR1] Adashek JA, Peaceman AM, Lopez-Zeno JA, Minogue JP, Socol ML (1993). Factors contributing to the increased cesarean birth rate in older parturient women. Am J Obstet Gynecol.

[CR2] Aguilar HN, Mitchell BF (2010). Physiological pathways and molecular mechanisms regulating uterine contractility. Hum Reprod Update.

[CR3] Al-Qahtani S, Heath A, Quenby S, Dawood F, Floyd R, Burdyga T, Wray S (2012). Diabetes is associated with impairment of uterine contractility and high caesarean section rate. Diabetologia.

[CR4] Arrowsmith S, Wray S, Quenby S (2011). Maternal obesity and labour complications following induction of labour in prolonged pregnancy. BJOG.

[CR5] Arrowsmith S, Quenby S, Weeks S, Burdyga T, Wray S (2012) Poor spontaneous and oxytocin-stimulated contractility in human myometrium from postdates pregnancies. PLoS One 7(5):e36787 10.1371/journal.pone.0036787 PMC3349652 22590608

[CR6] Batra S (1987). Increase by oestrogen of calcium entry and calcium channel density in uterine smooth muscle. Br J Pharmacol.

[CR7] Bell JS, Campbell DM, Graham WJ, Penney GC, Ryan M, Hall MH (2001). Do obstetric complications explain high caesarean section rates among women over 30?Retrospective analysis. BMJ.

[CR8] Bernal AL (2001). Overview of current research in parturition. Exp Physiol.

[CR9] Bianco A, Stone J, Lynch L, Lapinski R, Berkowitz G, Berkowitz RL (1996). Pregnancy outcome at age 40 and older. Obstet Gynecol.

[CR10] Bulletti C, de Ziegler D, Polli V, Diotallevi L, Del Ferro E, Flamigni C (2000). Uterine contractility during the menstrual cycle. Hum Reprod.

[CR11] Burger HG, Dudley E, Mamers P, Robertson D, Groome N, Dennerstein L (2002) The ageing female reproductive axis I. Novartis Found Symp 242:161-167; discussion 167–171 11855686

[CR12] Cnattingius R, Cnattingius S, Notzon FC (1998). Obstacles to reducing cesarean rates in a low-cesarean setting: the effect of maternal age, height, and weight. Obstet Gynecol.

[CR13] Crichton CA, Taggart MJ, Wray S, Smith GL (1993). Effects of pH and inorganic phosphate on force production in alpha-toxin-permeabilized isolated rat uterine smooth muscle. J Physiol.

[CR14] de Vries K, Lyons EA, Ballard G, Levi CS, Lindsay DJ (1990). Contractions of the inner third of the myometrium. Am J Obstet Gynecol.

[CR15] Domali E, Asprodini E, Molyvdas PA, Messinis IE (2001). In vitro effects of endothelin-1 on the contractility of myometrium obtained from pre- and postmenopausal women. J Endocrinol.

[CR16] Floyd R, Wray S (2007). Calcium transporters and signalling in smooth muscles. Cell Calcium.

[CR17] Garfield RE (1988). Classic illustration from Gap junctions: their presence and necessity in myometrium during parturition. By R. E. Garfield, et al., Science 1977. Eur J Obstet Gynecol Reprod Biol.

[CR18] Gimpl G, Fahrenholz F (2000) Human oxytocin receptors in cholesterol-rich vs. cholesterol-poor microdomains of the plasma membrane. Eur J Biochem/FEBS 267(9):2483–2497 10.1046/j.1432-1327.2000.01280.x 10785367

[CR19] Gimpl G, Burger K, Politowska E, Ciarkowski J, Fahrenholz F (2000) Oxytocin receptors and cholesterol: interaction and regulation. Exp Physiol 85 Spec No:41S–49S 10.1111/j.1469-445x.2000.tb00006.x 10795905

[CR20] Gordon D, Milberg J, Daling J, Hickok D (1991). Advanced maternal age as a risk factor for cesarean delivery. Obstet Gynecol.

[CR21] Greenberg MB, Cheng YW, Sullivan M, Norton ME, Hopkins LM, Caughey AB (2007) Does length of labor vary by maternal age? Am J Obstet Gynecol 197(4):428, e421–427. doi:10.1016/j.ajog.2007.06.058 10.1016/j.ajog.2007.06.058 17904990

[CR22] Heaton RC, Wray S, Eisner DA (1993). Effects of metabolic inhibition and changes of intracellular pH on potassium permeability and contraction of rat uterus. J Physiol.

[CR23] Heyn R, Makabe S, Correr S, Naguro T, Familiari G (2005). Pietro Motta’s latest studies on the microanatomy of menopause: revised concepts on the beauty of aging. Ital J Anat Embryol.

[CR24] Horowitz A, Menice CB, Laporte R, Morgan KG (1996). Mechanisms of smooth muscle contraction. Physiol Rev.

[CR25] Kido A, Togashi K, Nakai A, Kataoka M, Fujiwara T, Kataoka ML, Fujimoto R, Koyama T, Fujii S (2006). Investigation of uterine peristalsis diurnal variation. Magn Reson Imaging.

[CR26] Kirz DS, Dorchester W, Freeman RK (1985). Advanced maternal age: the mature gravida. Am J Obstet Gynecol.

[CR27] Kunz G, Leyendecker G (2002). Uterine peristaltic activity during the menstrual cycle: characterization, regulation, function and dysfunction. Reprod Biomed Online.

[CR28] Kupittayanant S, Luckas MJ, Wray S (2002). Effect of inhibiting the sarcoplasmic reticulum on spontaneous and oxytocin-induced contractions of human myometrium. BJOG.

[CR29] Le Ray C, Scherier S, Anselem O, Marszalek A, Tsatsaris V, Cabrol D, Goffinet F (2012). Association between oocyte donation and maternal and perinatal outcomes in women aged 43 years or older. Hum Reprod.

[CR30] Luckas MJ, Wray S (2000). A comparison of the contractile properties of human myometrium obtained from the upper and lower uterine segments. BJOG.

[CR31] Lyons EA, Taylor PJ, Zheng XH, Ballard G, Levi CS, Kredentser JV (1991). Characterization of subendometrial myometrial contractions throughout the menstrual cycle in normal fertile women. Fertil Steril.

[CR32] Main DM, Main EK, Moore DH (2000). The relationship between maternal age and uterine dysfunction: a continuous effect throughout reproductive life. Am J Obstet Gynecol.

[CR33] Maurer RR, Foote RH (1972). Maternal ageing and embryonic mortality in the rabbit. II. Hormonal changes in young and ageing females. J Reprod Fertil.

[CR34] Mulholland J, Jones CJ (1993). Characteristics of uterine aging. Microsc Res Tech.

[CR35] Nakai A, Togashi K, Yamaoka T, Fujiwara T, Ueda H, Koyama T, Kobayashi H, Kagimura T, Fujii S, Konishi J (2003). Uterine peristalsis shown on cine MR imaging using ultrafast sequence. J Magn Reson Imaging.

[CR36] Noble K, Zhang J, Wray S (2006). Lipid rafts, the sarcoplasmic reticulum and uterine calcium signalling: an integrated approach. J Physiol.

[CR37] Noble K, Arrowsmith S, Turton P, Quenby S, Wray S (2009) High myometrial cholesterol impairs uterine contractility. Reprod Sci (Suppl 3) 16:296A; abstract only

[CR38] Parkington HC, Tonta MA, Brennecke SP, Coleman HA (1999). Contractile activity, membrane potential, and cytoplasmic calcium in human uterine smooth muscle in the third trimester of pregnancy and during labor. Am J Obstet Gynecol.

[CR39] Price SA, Bernal AL (2001). Uterine quiescence: the role of cyclic AMP. Exp Physiol.

[CR40] Rigby SL, Barhoumi R, Burghardt RC, Colleran P, Thompson JA, Varner DD, Blanchard TL, Brinsko SP, Taylor T, Wilkerson MK, Delp MD (2001). Mares with delayed uterine clearance have an intrinsic defect in myometrial function. Biol Reprod.

[CR41] Schoen C, Rosen T (2009). Maternal and perinatal risks for women over 44––a review. Maturitas.

[CR42] Shmigol AV, Eisner DA, Wray S (1999). The role of the sarcoplasmic reticulum as a Ca2+ sink in rat uterine smooth muscle cells. J Physiol.

[CR43] Shmygol A, Noble K, Wray S (2007). Depletion of membrane cholesterol eliminates the Ca_2+_-activated component of outward potassium current and decreases membrane capacitance in rat uterine myocytes. J Physiol.

[CR44] Smith RD, Babiychuk EB, Noble K, Draeger A, Wray S (2005). Increased cholesterol decreases uterine activity: functional effects of cholesterol alteration in pregnant rat myometrium. Am J Physiol Cell Physiol.

[CR45] Smith GC, Cordeaux Y, White IR, Pasupathy D, Missfelder-Lobos H, Pell JP, Charnock-Jones DS, Fleming M (2008). The effect of delaying childbirth on primary cesarean section rates. PLoS Med.

[CR46] Szal SE, Repke JT, Seely EW, Graves SW, Parker CA, Morgan KG (1994). [Ca^2+^]i signaling in pregnant human myometrium. Am J Physiol.

[CR47] Taggart MJ, Menice CB, Morgan KG, Wray S (1997). Effect of metabolic inhibition on intracellular Ca^2+^, phosphorylation of myosin regulatory light chain and force in rat smooth muscle. J Physiol.

[CR48] Tezuka N, Ali M, Chwalisz K, Garfield RE (1995). Changes in transcripts encoding calcium channel subunits of rat myometrium during pregnancy. Am J Physiol.

[CR49] Thilander G, King GJ, Garfield RE (1993). Connexin43 and gap junction content in the porcine myometrium during the estrous cycle. Theriogenology.

[CR50] Togashi K (2007). Uterine contractility evaluated on cine magnetic resonance imaging. Ann N Y Acad Sci.

[CR51] Togashi K, Kawakami S, Kimura I, Asato R, Takakura K, Mori T, Konishi J (1993). Sustained uterine contractions: a cause of hypointense myometrial bulging. Radiology.

[CR52] Wray S (2007). Insights into the uterus. Exp Physiol.

[CR53] Wray S, Noble K (2008). Sex hormones and excitation–contraction coupling in the uterus: the effects of oestrous and hormones. J Neuroendocrinol.

[CR54] Wray S, Tofts PS (1986). Direct in vivo measurement of absolute metabolite concentrations using 31P nuclear magnetic resonance spectroscopy. Biochim Biophys Acta.

[CR55] Wray S, Kupittayanant S, Shmygol A, Smith RD, Burdyga T (2001). The physiological basis of uterine contractility: a short review. Exp Physiol.

[CR56] Wu X, Morgan KG, Jones CJ, Tribe RM, Taggart MJ (2008). Myometrial mechanoadaptation during pregnancy: implications for smooth muscle plasticity and remodelling. J Cell Mol Med.

[CR57] Yogev Y, Melamed N, Bardin R, Tenenbaum-Gavish K, Ben-Shitrit G, Ben-Haroush A (2010) Pregnancy outcome at extremely advanced maternal age. Am J Obstet Gynecol 203(6):558 e551–557. doi:10.1016/j.ajog.2010.07.039 10.1016/j.ajog.2010.07.039 20965486

[CR58] Zhang J, Bricker L, Wray S, Quenby S (2007). Poor uterine contractility in obese women. BJOG.

[CR59] Zhang J, Kendrick A, Quenby S, Wray S (2007). Contractility and calcium signaling of human myometrium are profoundly affected by cholesterol manipulation: implications for labor?. Reprod Sci.

[CR60] Ziadeh S, Yahaya A (2001). Pregnancy outcome at age 40 and older. Arch Gynecol Obstet.

